# Supplementary material for: A Sec-Dependent Effector from “Candidatus Phytoplasma ziziphi” Suppresses Plant Immunity and Contributes to Pathogenicity
Source: Biology (Basel). 2025 May 10;14(5):528. doi: 10.3390/biology14050528 (PMC12108763; doi:10.3390/biology14050528)
Supplement: Supplementary file 1 [file biology-14-00528-s001.zip › Supplementary figures.pdf]

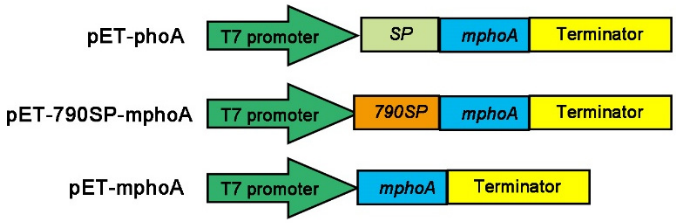

Figure S1. Schematic diagram of the prokaryotic expression cassettes for the phoA, mphoA and 790SP-mphoA. phoA: alkaline phosphatase. SP: signal peptide.

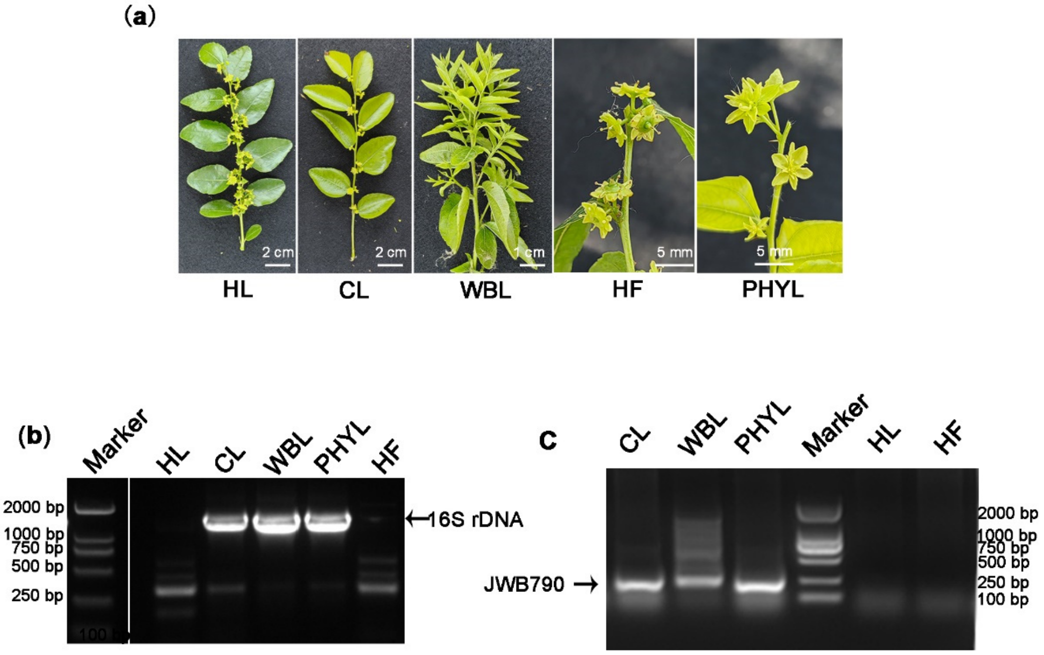

Figure S2. Detection of *Candidatus* Phytoplasma ziziphi and *JWB790* in tissues of *Ziziphus jujuba*. (**a**) Photos of healthy and phytoplasma-infected *Ziziphus jujuba* samples. HL: healthy leaves. CL: chlorotic leaves. WBL: witches'-broom leaves. HF: healthy flowers. PHYL: phyllody. (**b**) The presence of *Ca.* P. ziziphi in the infected trees was confirmed through the cloning of 16S ribosomal DNA. (**c**) The presence of JWB790 in the infected tissues was confirmed by PCR using the primer JWB790-F/R.

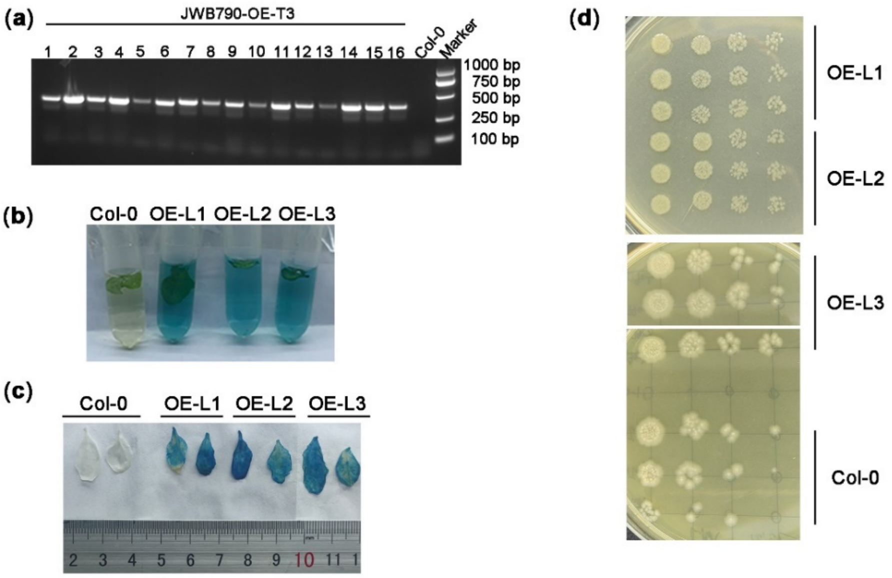

Figure S3. Detection of *JWB790* transgenic *Arabidopsis* plants and bacterial colonization analysis. (**a**) Molecular characterization of T_3 generation JWB790 over-expression (OE) transgenic *Arabidopsis thaliana* by PCR analysis with primers pBI121-F/R. (**b**) GUS (β-glucuronidase) activity visualized in *JWB790* transgenic plants 8 hours after X-Gluc incubation. (**c**) Representative leaf images following chlorophyll removal. (**d**) Detection of bacterial colonization in *JWB790* transgenic lines at 3 days post-inoculation (dpi). Dilutions were plated on rifampicin-supplemented Luria-Marine (LM) solid medium at 28℃ for 24-36 hours, with three technical replicates per sample.

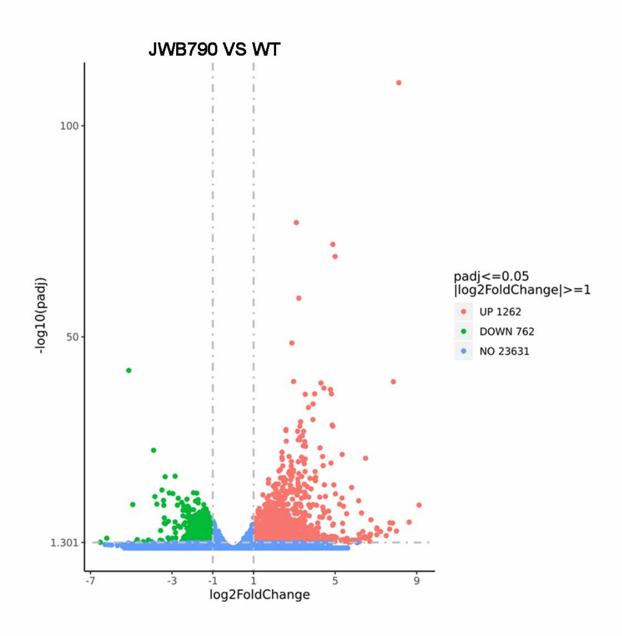

Figure S4. The volcano map of the differentially expressed genes (DEGs) between *JWB790* transgenic *Arabidopsis* plants and wild-type (WT) Col-0.
